# Factors related to loss to follow‐up and low compliance in *Helicobacter pylori*‐infected children: The EuroPedHp Registry

**DOI:** 10.1002/jpn3.70387

**Published:** 2026-02-24

**Authors:** Thu Giang Le Thi, Kallirroi Kotilea, José Cabral, Michal Kori, Maria Luz Cilleruelo, Marta Tavares, Josefa Barrio, Vaidotas Urbonas, Matjaž Homan, Zrinjka Misak, Nicolas Kalach, Pedro Urruzuno, Martina Klemenak, Andreas Krahl, Andrea Chiaro, Josef Sykora, Meltem Korkut Ugras, Jan de Laffolie, Erasmo Miele, Alexandra Papadopoulou, Sibylle Koletzko, for the Helicobacter pylori & Other Gastritis Special Interest Group of ESPGHAN, Thu Giang Le Thi, Kallirroi Kotilea, José Cabral, Michal Kori, Maria Luz Cilleruelo, Marta Tavares, Josefa Barrio, Vaidotas Urbonas, Matjaž Homan, Zrinjka Misak, Nicolas Kalach, Pedro Urruzuno, Martina Klemenak, Andreas Krahl, Andrea Chiaro, Josef Sykora, Meltem Korkut Ugras, Jan de Laffolie, Erasmo Miele, Alexandra Papadopoulou, Sibylle Koletzko

**Affiliations:** ^1^ Department of Pediatrics, Dr. von Hauner Children's Hospital LMU University Hospital Munich Munich Germany; ^2^ Stiftung Kindergesundheit, c/o Dr. von Hauner Children's Hospital LMU University Hospital Munich Munich Germany; ^3^ Université Libre de Bruxelles, Hôpital Universitaire des Enfants Reine Fabiola Brussels Belgium; ^4^ Child and Adolescent Centre, CUF Tejo Hospital Lisbon Portugal; ^5^ Pediatric Gastroenterology, Kaplan Medical Centre Rehovot Israel; ^6^ Faculty of Medicine Hebrew University of Jerusalem Jerusalem Israel; ^7^ Pediatrics Department. Gastroenterology Unit University Hospital Puerta de Hierro Majadahonda Madrid Spain; ^8^ Pediatric Gastroenterology Department Division of Pediatrics, Centro Materno Infantil do Norte, ICBAS ‐ Instituto de Ciências Biomédicas Abel Salazar Porto Portugal; ^9^ Pediatrics Department, Gastroenterology Unit University Hospital, Fuenlabrada Madrid Spain; ^10^ Clinic of Children's Diseases of Vilnius University Faculty of Medicine Vilnius Lithuania; ^11^ Department of Gastroenterology, Hepatology, and Nutrition University Children's Hospital, Faculty of Medicine, University of Ljubljana Ljubljana Slovenia; ^12^ Referral Centre for Pediatric Gastroenterology and Nutrition, Children's Hospital Zagreb University of Zagreb School of Medicine Zagreb Croatia; ^13^ Saint Antoine Pediatric Clinic, Saint Vincent de Paul Hospital, Groupement des Hôpitaux de l'Institut Catholique de Lille (GHICL) Catholic University Lille France; ^14^ Pediatric Gastroenterology Unit. Hospital 12 de Octubre Madrid Spain; ^15^ Gastroenterology, Hepatology and Nutrition Unit, Department of Paediatrics University Medical Centre Maribor Maribor Slovenia; ^16^ Darmstädter Kinderkliniken Prinzessin Margaret Darmstadt Germany; ^17^ Pediatric Gastroenterology and Endoscopy Unit, Institute Giannina Gaslini Genoa Italy; ^18^ Department of Paediatrics Charles University in Prague, Faculty of Medicine in Pilsen Czech Republic; ^19^ Department of Pediatrics, Gastroenterology Hepatology and Nutrition Yeditepe University Faculty of Medicine İstanbul Türkiye; ^20^ Department of General Pediatrics and Neonatology, Centre of Child and Adolescent Medicine Justus‐Liebig‐University Gießen Giessen Germany; ^21^ Department of Translational Medical Science University of Naples Federico II Naples Italy; ^22^ First Department of Paediatrics University of Athens, Agia Sophia Children's Hospital Athens Greece; ^23^ Department of Pediatrics, Gastroenterology and Nutrition School of Medicine Collegium Medicum University of Warmia and Mazury Olsztyn Poland

**Keywords:** anti‐*Helicobacter pylori* therapy, eradication, medication adherence, monitoring, paediatric

## Abstract

**Objectives:**

Monitoring for *Helicobacter pylori (H. pylori)*‐eradication is important, since symptom improvement does not indicate treatment success. Using EuroPedHp Registry data, we investigated characteristics of children missing monitoring visits after prescribed therapy, compliance effect on eradication, and factors associated with loss to follow‐up and low compliance.

**Methods:**

Between 2017 and 2020, 30 paediatric hospitals from 17 European countries reported 1605 children with biopsy‐proven *H. pylori*‐infection. Children with prescribed therapy were analysed. Risk factors for loss to follow‐up or low compliance (taking ≤90% of prescribed medications) were identified applying multivariable logistic regression.

**Results:**

Of 1263 infected children with prescribed therapy, 390 (31%) were lost to follow‐up. Risk factors for loss to follow‐up included nausea/dyspepsia (*p* = 0.004) or gastrointestinal bleeding (*p* = 0.03) as indication for endoscopy, living in Israel or Türkiye (*p* = 0.0002), and having no antibiotic susceptibility result (*p* = 0.004). Risk decreased with living in Southern Europe (*p* = 0.002), migration background (*p* = 0.052), and probiotic use during therapy (*p* = 0.02). Low compliance, reported in 69/831 (8%) children with follow‐up data, was associated with vomiting (*p* = 0.003), peptic ulcers or erosions (*p* = 0.03), living in EasternEurope (*p* = 0.009), Israel or Türkiye (*p* = 0.0008), and any adverse event during therapy (*p* = 0.0009). First‐line tailored triple therapy (TTT) for 14 days (*N* = 480) was successful in 92% with excellent versus 61% with low compliance (*p* < 0.0001). After ≥1 failed therapies (*N* = 60), TTT was successful in 71% with high versus 13% with low compliance (*p* = 0.003).

**Conclusion:**

The registry data identified several factors associated with non‐adherence to medication and monitoring visits. Improving information to patient/caregiver may increase adherence, care and treatment success.

## Abstract (highlights)

**What is Known:**

- Guidelines recommend monitoring *Helicobacter pylori* eradication post‐therapy, as symptom improvement does not guarantee treatment success.
- Poor medication compliance is an independent risk factor for eradication failure, regardless of treatment regimen.

**What is New:**

- Loss to follow‐up occurred in 31% of 1263 children with prescribed therapy and was related to nausea/dyspepsia or gastroduodenal bleeding indicating endoscopy, living in certain countries, and lack of antibiotic susceptibility results.
- Low compliance, reported in 8% of children with follow‐up data, was associated with vomiting, peptic ulcers/erosions, certain treatment regimens, and adverse events.
- Knowledge of risk factors for non‐adherence to monitoring or drug intake allows actions to improve care.

## Graphical abstract

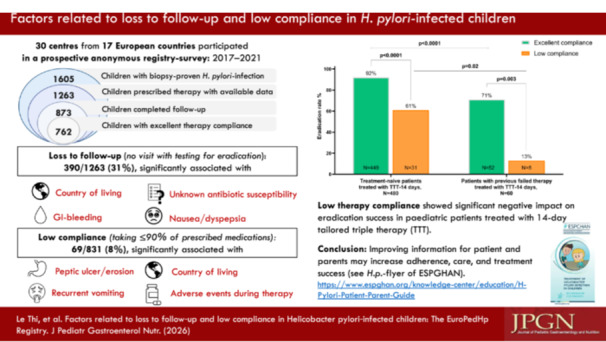

## 1 INTRODUCTION

*Helicobacter pylori (H. pylori)* is one of the most common bacterial pathogens in humans worldwide.^1^, ^2^ The infection causes chronic active gastritis and increases the lifetime risk for gastroduodenal peptic ulcers and gastric malignancies.^3^ *H. pylori*‐infection is predominantly acquired in children under 6 years of age^4^ and often remains asymptomatic during childhood.^5^, ^6^ Eradication of *H. pylori‐*infection improves gastric inflammation and reduces the risk for recurrent peptic ulcer disease (PUD) and malignancies.^7^ The goal of anti‐*H. pylori* therapy is a primary eradication rate of at least 90%.^8^, ^9^, ^10^, ^11^, ^12^ Treatment failure may result from inadequate dosage, insufficient therapy duration, infecting strains not susceptible to prescribed antibiotics, and lack of treatment adherence.^13^, ^14^, ^15^ Poor therapy compliance is an independent risk factor for eradication failure, regardless of treatment regimens.^14^, ^16^ Children taking less than 90% of prescribed drugs over 14 days had nearly six times higher risk of treatment failure to first‐line therapy than those with excellent compliance.^16^

Monitoring for bacterial clearance 6–8 weeks after completion of anti‐*H. pylori* therapy is strongly recommended, regardless of any change in symptoms.^10^, ^17^, ^18^, ^19^ Follow‐up eradication testing in paediatric patients can be performed noninvasively using ¹³C urea breath testing or monoclonal stool antigen assays to detect *H. pylori* infection.^10^, ^11^, ^17^ Nevertheless, eradication testing is frequently not performed after treatment in both paediatric and adult populations.^15^, ^16^, ^17^, ^20^, ^21^

The European Society for Paediatric Gastroenterology, Hepatology, and Nutrition (ESPGHAN) Working Group on *H. pylori* infection established the EuroPedHp‐Registry to collect real‐world data from *H. pylori*‐infected children to identify deficiencies and improve guidance for the management of *H. pylori*‐infected paediatric patients. We used registry data to identify factors associated with loss to follow‐up and low therapy compliance in *H. pylori‐*infected children, and to evaluate eradication success of the 14‐day tailored triple therapy (TTT) in relation to compliance.

## 2 METHODS

### 2.1 Ethics statement

We retrieved irreversibly anonymized data of paediatric patients with endoscopically confirmed *H. pylori‐*infection from the EuroPedHp‐Registry (2017–2020).^16^ The Ethical Committee at the LMU University Hospital Munich, Germany, approved the survey protocol for anonymized patient data without requiring individual written consent from patients/caregivers (project number: 105‐13).^16^ This registry was financially supported by ESPGHAN and research funds from Prof. Dr. med. Sibylle Koletzko at LMU University Hospital Munich, Germany.

### 2.2 Data selection

To evaluate factors associated with loss to follow‐up, we included all paediatric patients diagnosed between January 1, 2017 to December 31, 2020 with biopsy‐confirmed *H. pylori*‐infection (histopathology plus culture and/or reverse transcription polymerase chain reaction [RT‐PCR]) and available information on prescribed treatment. Patients without monitoring for bacterial clearance were considered as “lost to follow‐up.” Children returning for monitoring visits post‐therapy were assessed for intake of prescribed drugs and formed a subgroup to analyse factors associated with low therapy compliance, defined as taking ≤90% of prescribed medications.

Data on patient characteristics include demographics, gastrointestinal symptoms as indications for endoscopy, comorbidities, macroscopic findings, prescribed treatment, and outcomes. Based on residency and birthplace, we categorised countries of living and countries of birth into European regions (Northern, Western, Southern, Eastern), Israel, Türkiye, and other regions (Supporting Information S1: File S1).^16^, ^22^ Immigration status was assigned if patient or parent(s) were born outside country of living. Alarm signs and symptoms as primary endoscopy indications were defined as anaemia, upper gastrointestinal bleeding, weight loss, chronic diarrhoea, dysphagia, and recurrent vomiting. Non‐alarm symptoms included abdominal pain, dyspepsia, nausea, bloating, and constipation.^22^, ^23^

Clinicians were encouraged to follow ESPGHAN/NASPGHAN (North American Society for Pediatric Gastroenterology, Hepatology, and Nutrition) guidelines updated in 2016 for managing *H. pylori*‐infection in children and adolescents.^11^ Successful eradication was confirmed by negative results of ^13^C‐urea breath test, monoclonal faecal antigen test, or histopathology and culture after repeated gastric biopsies. Eradication success is significantly associated with antibiotic regimen and therapy duration.^16^, ^21^ To compare eradication success between patients with high versus low compliance, we included only those receiving the guideline‐conform treatment regimen: a 14‐day TTT with proton pump inhibitors, amoxicillin, metronidazole (PAM), or clarithromycin (PAC),^11^ and excluded all patients with heterogeneous regimen, shorter therapy duration, or unknown antibiotic susceptibility results.

### 2.3 Statistical analysis

Descriptive statistics were used to summarise patient characteristics. Factors associated with loss to follow‐up were identified using multivariable logistic regression analyses. Based on patients’ characteristics, factors with a high likelihood of loss to follow‐up (*p* ≤ 0.25) were included in the multivariable logistic regression. Using backward elimination adjusted for gender, age (years), and country of living, the final logistic regression with no missing co‐variates was presented with estimated odds ratio (OR), 95% confidence interval (CI), and *p*‐values from Wald‐chi‐square test. The same approach was applied to identify factors associated with low compliance.

A sensitivity analysis examined factors associated with loss to follow‐up and low compliance including only treatment‐naïve patients, comprising 85%–87% of *H. pylori*‐infected children reported in the total cohort.^16^, ^21^ A post‐hoc analysis investigated the link between adverse events and compliance in children receiving high and standard amoxicillin dose regimens according to the updated guidelines.^11^

Data were analysed using SAS Enterprise Guide 8.1 (Statistical Analysis Software, SAS Institute Inc, USA) and Prism 10.2.1 (GraphPad Software, USA).

## 3 RESULTS

Of 1605 paediatric patients in the EuroPedHp registry (2017–2020), 1263 were prescribed therapy and met the inclusion criteria for the follow‐up analysis. Among them, 831 children with complete follow‐up data were included in the compliance analysis. (Supporting Information S1: File S2).

### 3.1 Characteristics of children lost to follow‐up compared to those with monitoring visits

Of 1263 infected patients with prescribed therapy, 53% were female, and the median age was 13.2 years (Table 1). Patients lost to follow‐up (390/1263, 31%) were slightly older, more likely living in Israel or Türkiye or born outside Europe. Loss to follow‐up was lower in immigrants (24%, *N* = 340) than in non‐immigrant children (31%, *N* = 689) (*p* = 0.02). Compared to those with completed monitoring, they reported more often dyspepsia (37% vs. 30%, *p* = 0.04) and GI bleeding (45% vs. 30%, *p* = 0.049) and had a high rate of unavailable/unknown antibiotic susceptibility results (42%, *N* = 195, *p* < 0001). Approximately 35% of patients treated with PAC for 14 days were lost to follow‐up (*N* = 519), versus 28% with PAM for 14 days (*N* = 458) and 19% receiving sequential therapy for 10 days (*N* = 106) (*p* < 0.006). Children receiving probiotics along with their prescribed therapy were less likely to miss monitoring than those who did not (23% vs. 31%, *p* = 0.005) (Table 1).

**Table 1:** Loss to follow‐up among paediatric patients with prescribed therapy for *Helicobacter pylori* infection, *N* = 1263.

Factors *N* (%, row percent)	Total *N* = 1263	Loss to follow‐up *N* = 390 (31%)	Follow‐up completed *N* = 873 (69%)	*p*‐value^a^
Demographics				
Gender				0.75
Male	588	179 (30%)	409 (70%)	
Female	675	211 (31%)	464 (69%)	
Age (years), median (IQR)	13.2 (10.2, 15.5)	13.6 (10.0, 16.3)	13.0 (10.3, 15.2)	**0.04**
Weight (kg), *N* = 1244				**0.01**
<25	143	58 (41%)	85 (59%)	
25‐34	191	50 (26%)	141 (74%)	
>35	910	273 (30%)	637 (70%)	
Country of living^b^				**<0.001**
Northern/Western Europe	331	99 (30%)	232 (70%)	
Southern Europe	539	120 (22%)	419 (78%)	
Eastern Europe	263	99 (38%)	164 (62%)	
Israel & Turkey	130	72 (55%)	58 (45%)	
Country of birth of patient,^b^ *N* = 1120				**<0.001**
Northern/Western Europe	226	53 (23%)	173 (77%)	
Southern Europe	415	98 (24%)	317 (76%)	
Eastern Europe	269	94 (35%)	175 (65%)	
Israel & Turkey	131	71 (54%)	60 (46%)	
Outside of Europe	79	27 (34%)	52 (66%)	
Country of birth of mother,^b^ *N* = 1014				**<0.001**
Northern/Western Europe	53	18 (34%)	35 (66%)	
Southern Europe	341	81 (24%)	260 (76%)	
Eastern Europe	262	73 (28%)	189 (72%)	
Israel & Turkey	126	66 (52%)	60 (48%)	
Outside of Europe	232	57 (25%)	175 (75%)	
Migration background,^c^ *N* = 1029				**0.02**
Immigrant	340	82 (24%)	258 (76%)	
Non‐immigrant	689	215 (31%)	474 (69%)	
Medical history				
Diagnosed with comorbidities, *N* = 1249				0.80
GI disorders	126	37 (29%)	89 (71%)	
Non‐GI disorders	139	46 (33%)	93 (67%)	
No comorbidity	984	304 (31%)	680 (69%)	
Previous *H. pylori* eradication therapy				1.00
Yes	191	59 (31%)	132 (69%)	
No	1072	331 (31%)	741 (69%)	
Non‐alarm GI symptoms				
Report on any non‐alarm GI symptoms				0.24
Yes	997	300 (30%)	697 (70%)	
No	266	90 (34%)	176 (66%)	
Abdominal pain, *N* = 1258				0.19
Yes	950	283 (30%)	667 (70%)	
No	308	104 (34%)	204 (66%)	
Dyspepsia incl. nausea, *N* = 1258				**0.04**
Yes	216	79 (37%)	137 (63%)	
No	1042	308 (30%)	734 (70%)	
Bloating, *N* = 1258				0.92
Yes	67	21 (31%)	46 (69%)	
No	1191	366 (31%)	825 (69%)	
Constipation, *N* = 1258				0.65
Yes	40	11 (28%)	29 (73%)	
No	1218	376 (31%)	842 (69%)	
Alarm symptoms and signs^d^				
Any alarm symptoms and signs				0.13
Yes	343	117 (34%)	226 (66%)	
No	920	273 (30%)	647 (70%)	
Anemia				0.14
Yes	58	23 (40%)	35 (60%)	
No	1205	367 (30%)	838 (70%)	
Chronic diarrhoea				0.98
Yes	49	15 (31%)	34 (69%)	
No	1209	372 (31%)	837 (69%)	
Dysphagia				0.54
Yes	13	3 (23%)	10 (77%)	
No	1250	387 (31%)	863 (69%)	
GI‐bleeding				**0.049**
Yes	40	18 (45%)	22 (55%)	
No	1223	372 (30%)	851 (70%)	
Recurrent vomiting				0.29
Yes	209	71 (34%)	138 (66%)	
No	1054	319 (30%)	735 (70%)	
Macroscopic findings				
Antral nodularity, *N* = 1245				0.39
Yes	1056	319 (30%)	737 (70%)	
No	189	63 (33%)	126 (67%)	
Peptic ulcers, *N* = 1246				0.52
Yes	90	25 (28%)	65 (72%)	
No	1156	359 (31%)	797 (69%)	
Erosions, *N* = 1245				0.97
Yes	204	63 (31%)	141 (69%)	
No	1041	320 (31%)	721 (69%)	
Antibiotic susceptibility testing^e^				
Susceptibility groups				**<0.001**
MET‐S/CLA‐S	566	160 (28%)	406 (72%)	
MET‐S/CLA‐R	195	43 (22%)	152 (78%)	
MET‐R/CLA‐S	212	77 (36%)	135 (64%)	
MET‐R/CLA‐R	95	28 (29%)	67 (71%)	
Culture negative/results not available	195	82 (42%)	113 (58%)	
Therapy				
Common treatment regimens				**0.006**
PPI + AMO + CLA (14 days)	519	180 (35%)	339 (65%)	
PPI + AMO + MET (14 days)	458	130 (28%)	328 (72%)	
Sequential therapy (10 days)	106	20 (19%)	86 (81%)	
Others^f^	180	60 (33%)	120 (67%)	
Daily intake frequency, *N* = 1147				0.29
Twice a day	938	291 (31%)	647 (69%)	
Three times a day	209	57 (27%)	152 (73%)	
Use of probiotics, *N* = 1259				**0.005**
Yes	203	47 (23%)	156 (77%)	
No	981	309 (31%)	672 (69%)	

*Note*: Results are presented in median and IQR from 25% quartile to 75% quartile for continuous variables and in frequency (*n*) and row percentage (%) for categorical variables. The bold values indicate the statistical significance of *p* < 0.05.

Abbreviations: AMO, amoxicillin; BMI, body mass index; CLA, clarithromycin; GI, gastrointestinal; *H. pylori, Helicobacter pylori*; IQR, interquartile range; MET, metronidazole; MET‐R/CLA‐R, strains resistant to both metronidazole and clarithromycin; MET‐R/CLA‐S, strains resistant to metronidazole but susceptible to clarithromycin; MET‐S/CLA‐R, strains susceptible to metronidazole but resistant to clarithromycin; MET‐S/CLA‐S, strains susceptible to both metronidazole and clarithromycin; PAC, treatment regimen with proton pump inhibitor, amoxicillin, clarithromycin; PAM, treatment regimen with proton pump inhibitor, amoxicillin, metronidazole; PCR, polymerase chain reaction; PPI, proton pump inhibitor; RT‐PCR, real‐time polymerase chain reaction; SEQ, sequential therapy.

^a^ *p*‐values obtained by Mann–Whitney‐*U*‐test for continuous variables, while Pearson's Chi‐square test for categorical variables as appropriate.

^b^ Country distribution was given in Supporting Information S1: File S1.

^c^ Immigrant patient was identified if this patient and/or his/her parents were born outside of country of living.

^d^ Symptoms were classified as alarm symptoms according to Thakka et al. 2014 (reference^22^ in main manuscript) and assessed using data regarding symptoms associated with *H. pylori* infection, primary indication for endoscopy, and additional details provided in the questionnaire by physicians.

^e^ Data were also collected from RT‐PCR test for clarithromycin susceptibility.

^f^ Other treatment regimens including PPI+ other antibiotic, PPI + CLA + MET, PPI + AMO + other than CLA or MET, PPI + AMO + CLA + MET, or PPI + antibiotic + Bismuth

### 3.2 Factors associated with loss to follow‐up

**Figure 1:**
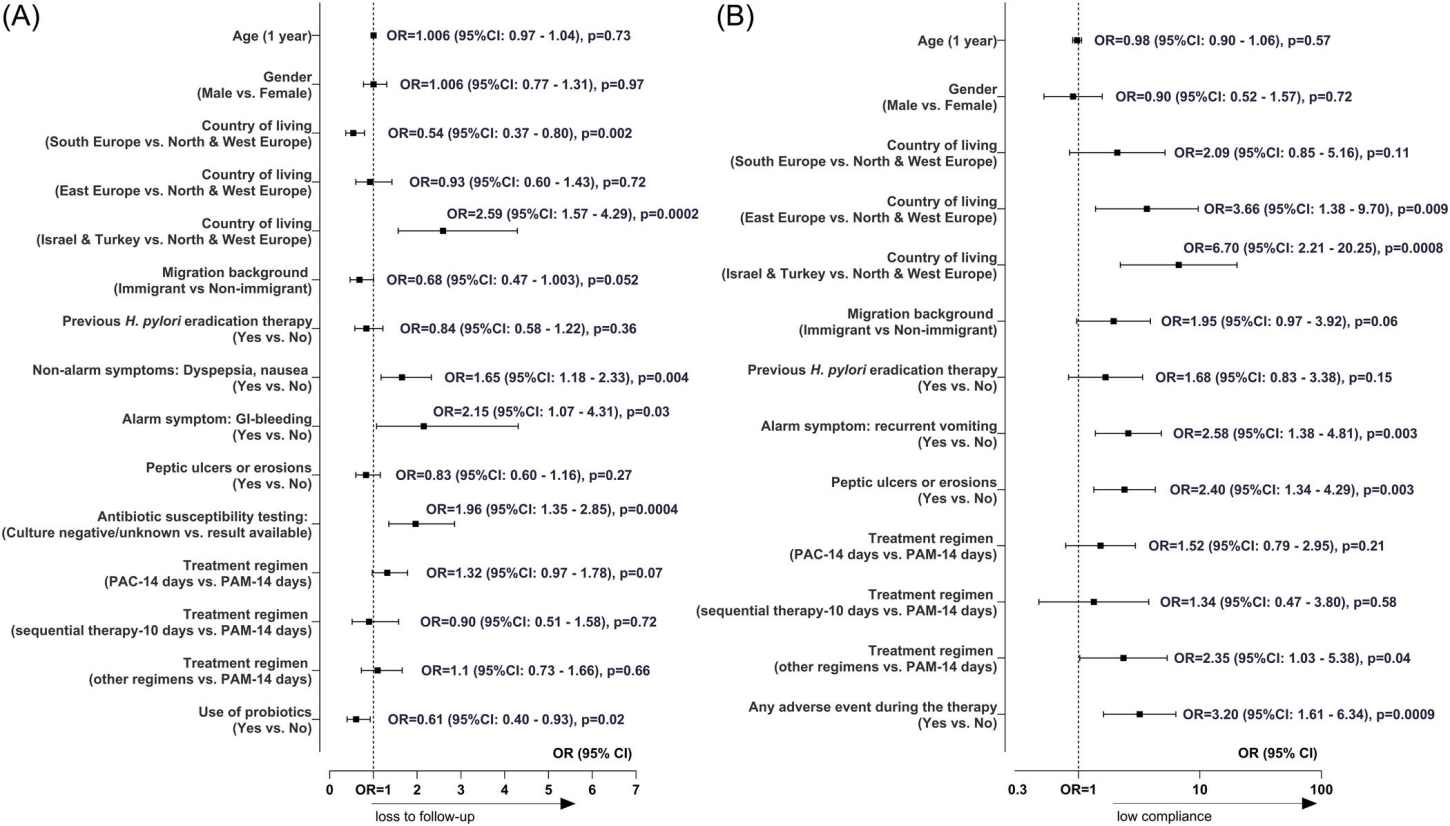
Factors associated (A) with loss to follow‐up among paediatric patients with prescribed therapy for *Helicobacter pylori* infection, *N* = 1263, and (B) with low compliance among paediatric patients treated for *H. pylori* infection, *N* = 831. *Note*: OR with 95% CIs were obtained from the final multivariable logistic regression adjusted for gender, age (years), and country of living. *p*‐values were determined using the Wald Chi‐square test to assess the significance of the OR. CI, confidence interval; GI, gastrointestinal; *H. pylori, Helicobacter pylori;* MET‐R/CLA‐R, strains resistant to both metronidazole and clarithromycin; MET‐R/CLA‐S, strains resistant to metronidazole but susceptible to clarithromycin; MET‐S/CLA‐R, strains susceptible to metronidazole but resistant to clarithromycin; MET‐S/CLA‐S, strains susceptible to both metronidazole and clarithromycin; OR, odd ratio; PAC, treatment regimen with proton pump inhibitor, amoxicillin, clarithromycin; PAM, treatment regimen with proton pump inhibitor, amoxicillin, metronidazole.

The results of the final multivariable logistic regression indicating factors associated with loss to follow‐up are presented in Figure 1A. Compared to children from Northern/Western Europe, those living in Israel or Türkiye had a 2.59‐times higher risk of loss to follow‐up (*p* = 0.0002), while those in Southern Europe had 0.54 times the odds (*p* = 0.03). Patients reporting dyspepsia and nausea had a 1.65 times higher risk (*p* = 0.004), and those with gastrointestinal bleeding had a 2.15 times higher risk for loss to follow‐up (*p* = 0.03) (Figure 1A). Notably, children with migration background had a 32% lower risk of missing follow‐up visits (*p* = 0.052). Children with unavailable or unknown antibiotic susceptibility results were twice as likely to miss monitoring visits (*p* = 0.0004). Children treated with 14‐day PAC had a trend toward higher risk for loss to follow‐up compared to those on 14‐day PAM (*p* = 0.07). Concurrent use of probiotics with prescribed therapy suggested a nearly 40% lower risk of loss to follow‐up (*p* = 0.02) (Figure 1A).

Sensitivity analysis of 1052 treatment‐naïve patients revealed a pattern similar to that observed in the total cohort of 1263 patients, with minimal deviations in the risk estimates (Supporting Information S1: File S3).

### 3.3 Characteristics of children with low versus excellent compliance

Therapy compliance among 831 children monitored for eradication success, including 69 (8%) reporting low compliance, are presented in Table 2. Low compliance was more frequent among children living in Israel and Türkiye compared to those living in Northern/Western Europe (29% vs. 6%, respectively; *p* < 0.001) (Table 2). Reporting only non‐alarm symptoms as indication for endoscopy was not related to therapy compliance, while patients with dysphagia or recurrent vomiting, or having peptic ulcers or erosions at endoscopy had significantly higher rates of low compliance (Table 2). Therapy compliance did not significantly differ across treatment regimens. However, only 6% of children with successful bacterial clearance (*N* = 701) exhibited low compliance compared to 23% (*N* = 130) who failed eradication (*p* < 0.0001). Children reporting adverse events during therapy had lower compliance (21%) than those who did not (7%) (*p* < 0.001). The most frequently reported adverse events included diarrhoea, metallic taste, dyspepsia, nausea, and recurrent vomiting (Table 2).

**Table 2:** Therapy compliance among paediatric patients treated for *Helicobacter pylori* infection, *N* = 831.

Factors *N* (%, row percent)	Total *N* = 831	Low compliance *N* = 69 (8%)	Excellent compliance *N* = 762 (92%)	*p*‐value^a^
Demographics				
Gender				0.63
Male	384	30 (8%)	354 (92%)	
Female	447	39 (9%)	408 (91%)	
Age (years), median (IQR)	13.0 (10.3, 15.1)	12.9 (10.0, 15.2)	13.0 (10.3, 15.1)	0.92
Weight (kg), *N* = 823				0.87
<25	82	8 (10%)	74 (90%)	
25–34	138	12 (9%)	126 (91%)	
>35	603	49 (8%)	554 (92%)	
Country of living^b^				**<0.001**
Northern/Western Europe	216	14 (6%)	202 (94%)	
Southern Europe	401	23 (6%)	378 (94%)	
Eastern Europe	162	17 (10%)	145 (90%)	
Israel & Turkey	52	15 (29%)	37 (71%)	
Country of birth of patient,^b^ *N* = 747				**<0.001**
Northern/Western Europe	167	10 (6%)	157 (94%)	
Southern Europe	306	20 (7%)	286 (93%)	
Eastern Europe	173	18 (10%)	155 (90%)	
Israel & Turkey	54	15 (28%)	39 (72%)	
Outside of Europe	47	6 (13%)	41 (87%)	
Country of birth of mother,^b^ *N* = 692				**<0.001**
Northern/Western Europe	34	3 (9%)	31 (91%)	
Southern Europe	252	14 (6%)	238 (94%)	
Eastern Europe	185	19 (10%)	166 (90%)	
Israel & Turkey	56	15 (27%)	41 (73%)	
Outside of Europe	165	15 (9%)	150 (91%)	
Migration background,^c^ *N* = 704				0.65
Immigrant	245	25 (10%)	220 (90%)	
Non‐immigrant	459	42 (9%)	417 (91%)	
Medical history				
Diagnosed comorbidities, *N* = 821				0.23
GI disorders	86	8 (9%)	78 (91%)	
Non‐GI disorders	86	3 (3%)	83 (97%)	
No comorbidity	649	57 (9%)	592 (91%)	
Previous *H. pylori* eradication therapy				**0.006**
Yes	123	18 (15%)	105 (85%)	
No	708	51 (7%)	657 (93%)	
Non‐alarm GI symptoms				
Any non‐alarm GI symptoms				0.97
Yes	661	55 (8%)	606 (92%)	
No	170	14 (8%)	156 (92%)	
Abdominal pain				0.91
Yes	632	53 (8%)	579 (92%)	
No	197	16 (8%)	181 (92%)	
Dyspepsia incl. nausea, *N* = 829				0.08
Yes	131	16 (12%)	115 (88%)	
No	698	53 (8%)	645 (92%)	
Bloating, *N* = 829				0.07
Yes	45	7 (16%)	38 (84%)	
No	784	62 (8%)	722 (92%)	
Constipation, *N* = 829				0.35
Yes	28	1 (4%)	27 (96%)	
No	801	68 (8%)	733 (92%)	
Alarm symptoms and signs^d^				
Any alarm symptoms and sign				**0.001**
Yes	214	29 (14%)	185 (86%)	
No	617	40 (6%)	577 (94%)	
Anemia				0.60
Yes	34	2 (6%)	32 (94%)	
No	797	67 (8%)	730 (92%)	
Chronic diarrhoea				0.15
Yes	33	5 (15%)	28 (85%)	
No	798	64 (8%)	734 (92%)	
Dysphagia				**0.006**
Yes	9	3 (33%)	6 (67%)	
No	822	66 (8%)	756 (92%)	
GI‐bleeding				0.36
Yes	22	3 (14%)	19 (86%)	
No	809	66 (8%)	743 (92%)	
Recurrent vomiting				**<0.001**
Yes	130	21 (16%)	109 (84%)	
No	701	48 (7%)	653 (93%)	
Macroscopic findings				
Antral nodularity, *N* = 821				0.96
Yes	702	58 (8%)	644 (92%)	
No	119	10 (8%)	109 (92%)	
Peptic ulcers, *N* = 820				**0.02**
Yes	61	10 (16%)	51 (84%)	
No	759	58 (8%)	701 (92%)	
Erosions, *N* = 820				**<0.001**
Yes	133	22 (17%)	111 (83%)	
No	687	46 (7%)	641 (93%)	
Antibiotic susceptibility testing^e^				
Susceptibility groups				0.09
MET‐S/CLA‐S	385	22 (6%)	363 (94%)	
MET‐S/CLA‐R	145	12 (8%)	133 (92%)	
MET‐R/CLA‐S	130	14 (11%)	116 (89%)	
MET‐R/CLA‐R	63	7 (11%)	56 (89%)	
Culture negative/results not available	108	14 (13%)	94 (87%)	
Therapy				
Common treatment regimens				0.23
PPI + AMO + CLA (14 days)	319	28 (9%)	291 (91%)	
PPI + AMO + MET (14 days)	317	21 (7%)	296 (93%)	
Sequential therapy (10 days)	85	6 (7%)	79 (93%)	
Others^f^	110	14 (13%)	96 (87%)	
Daily intake frequency, *N* = 759				0.39
Twice a day	613	51 (8%)	562 (92%)	
Three times a day	146	9 (6%)	137 (94%)	
Use of probiotics, *N* = 790				0.73
Yes	155	14 (9%)	141 (91%)	
No	635	53 (8%)	582 (92%)	
Eradication success				**<0.0001**
Yes	701	39 (6%)	662 (94%)	
No	130	30 (23%)	100 (77%)	
Adverse events during therapy, *N* = 797				
Any adverse event during therapy				**<0.001**
Yes	95	20 (21%)	75 (79%)	
No	702	47 (7%)	655 (93%)	
Abdominal pain				0.07
Yes	53	8 (15%)	45 (85%)	
No	744	59 (8%)	685 (92%)	
Diarrhoea				**0.008**
Yes	27	6 (22%)	21 (78%)	
No	770	61 (8%)	709 (92%)	
Metallic taste				**<0.001**
Yes	14	6 (43%)	8 (57%)	
No	783	61 (8%)	722 (92%)	
Dyspepsia incl. nausea				**0.04**
Yes	26	5 (19%)	21 (81%)	
No	771	62 (8%)	709 (92%)	
Recurrent vomiting				**<0.001**
Yes	22	8 (36%)	14 (64%)	
No	775	59 (8%)	716 (92%)	

*Note*: Results were presented in median and IQR from 25% quartile to 75% quartile for continuous variables and in frequency (*n*) and row percentage (%) for categorical variables. The bold values indicate the statistical significance of *p* < 0.05.

Abbreviations: AMO, amoxicillin; BMI, body mass index; CLA, clarithromycin; GI, gastrointestinal; *H. pylori, Helicobacter pylori;* IQR, interquartile range; MET, metronidazole; MET‐R/CLA‐R, strains resistant to both metronidazole and clarithromycin; MET‐R/CLA‐S, strains resistant to metronidazole but susceptible to clarithromycin; MET‐S/CLA‐R, strains susceptible to metronidazole but resistant to clarithromycin; MET‐S/CLA‐S, strains susceptible to both metronidazole and clarithromycin; PAC, treatment regimen with proton pump inhibitor, amoxicillin, clarithromycin; PAM, treatment regimen with proton pump inhibitor, amoxicillin, metronidazole; PCR, polymerase chain reaction; PPI, proton pump inhibitor; RT‐PCR, real‐time polymerase chain reaction; SEQ, sequential therapy.

^a^ *p*‐Values obtained by Mann–Whitney‐*U*‐test for continuous variables, while Pearson's Chi‐square test for categorical variables as appropriate.

^b^ Country distribution was given in Supporting Information S1: File S1.

^c^ Immigrant patient was identified if this patient and/or his/her parents were born outside of country of living.

^d^ Symptoms were classified as alarm symptoms according to Thakka et al. 2014 (reference ^22^ in main manuscript) and assessed using data regarding symptoms associated with *H. pylori* infection, primary indication for endoscopy, and additional details provided in the questionnaire by physicians.

^e^ Data were also collected from RT‐PCR test for clarithromycin susceptibility.

^f^ Other treatment regimens including PPI+ other antibiotics, PPI + CLA + MET, PPI + AMO + other than CLA or MET, PPI + AMO + CLA + MET, or PPI + antibiotic + Bismuth.

### 3.4 Factors associated with low compliance

Figure 1B illustrates multivariate analysis results investigating factors associated with low compliance with anti‐*H. pylori* therapy. Children living in Israel and Türkiye had a 6.7 times increased risk for low compliance versus those living in Northern and Western Europe (*p* = 0.0008) (Figure 1B). Immigrant children tended to have nearly twice the risk for low compliance (*p* = 0.06). Low compliance was significantly associated with recurrent vomiting and the presence of peptic ulcers or erosions (Figure 1B). Adverse events during therapy, such as diarrhoea, recurrent vomiting, dyspepsia, nausea, or metallic taste, tripled the risk of low compliance (*p* = 0.0009) (Figure 1B).

Sensitivity analysis of 708 treatment‐naïve patients confirmed the association between region of living, recurrent vomiting, presence of peptic ulcers or erosions at endoscopy, and experiencing adverse events during therapy with low compliance by higher risk estimates (Supporting Information S1: File S4). Migration background was a significant factor associated with nearly three times the risk for low compliance (*p* = 0.01) (Supporting Information S1: File S4).

### 3.5 Effect of compliance on eradication success in children treated with TTT for 14 days

**Figure 2:**
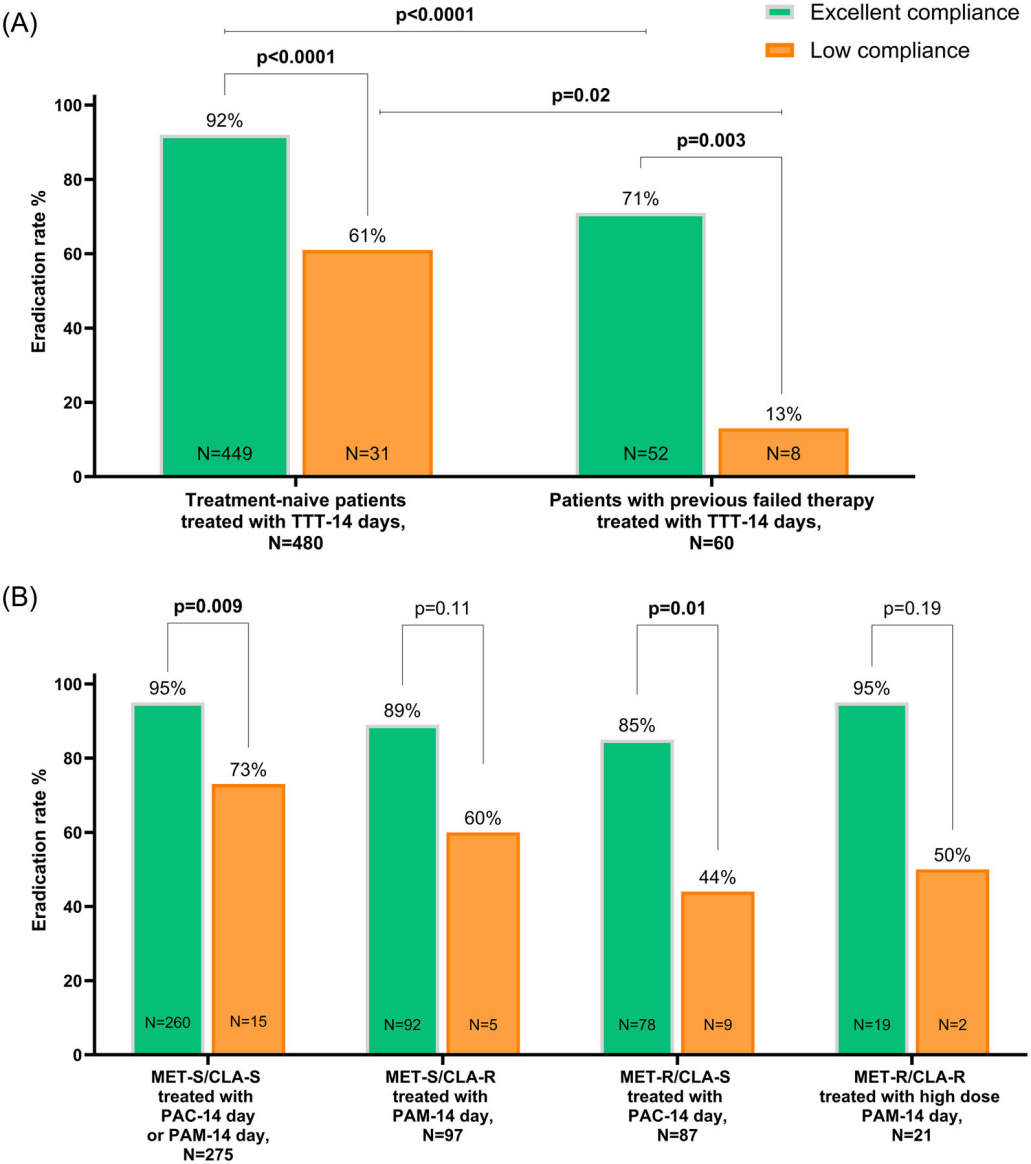
Effect of excellent versus low therapy compliance on eradication success in *Helicobacter pylori* infected paediatric patients treated with TTT for 14 days: (A) Eradication rate in treatment naïve patients (left) and children with previous failed therapy (right), *N* = 540, and (B) Eradication rate in treatment‐naïve paediatric patients treated with PAC or PAM according to antibiotic susceptibility results, *N* = 480. *Note*: ER% represents ER in percent (%) as the proportion of all patients treated successfully with a confirmed negative test after completed treatment relative to all patients treated in a specific sub‐group. *p*‐Values were obtained from Fisher‐exact‐test to determine the significant difference in ER between the patient groups with excellent compliance versus those with low compliance. ER, eradication rate; MET‐R/CLA‐R, strains resistant to both metronidazole and clarithromycin; MET‐R/CLA‐S, strains resistant to metronidazole but susceptible to clarithromycin; MET‐S/CLA‐R, strains susceptible to metronidazole but resistant to clarithromycin; MET‐S/CLA‐S, strains susceptible to both metronidazole and clarithromycin; ns, not significant; PAC, treatment regimen with proton pump inhibitor, amoxicillin, clarithromycin; PAM, treatment regimen with proton pump inhibitor, amoxicillin, metronidazole; TTT, 2‐week tailored triple therapy.

We found a highly significant negative impact of low therapy compliance on eradication success in paediatric patients, both treatment‐naïve and those after treatment failure. First‐line 14‐day TTT (*N* = 480) achieved 92% eradication with excellent versus 61% with low compliance (*p* < 0.0001). Among children with previous failed therapy (*N* = 60), TTT was successful in 71% with excellent versus 13% with low compliance (*p* = 0.003) (Figure 2A). Despite prescribing a 14‐day TTT, eradication rates were significantly lower in patients with previous failed therapy compared to treatment naïve children regardless of compliance. In treatment‐naïve children, the difference in eradication rates between excellent and low compliant patients is lowest if the *H. pylori* strain was fully susceptible (∆ = 22%) and highest in patients infected with strains resistant to both clarithromycin and metronidazole (∆ = 45%) (Figure 2B). The significance levels were not reached in all subgroup comparisons due to small sample sizes.

### 3.6 Effect of high versus standard dose amoxicillin regimen on eradication success concerning adverse events during therapy and compliance

The ESPGHAN/NASPGHAN guidelines 2016^11^ recommended a 14‐day TTT using high‐dose amoxicillin (maximal 3 g in three divided doses) instead of standard dose (maximal 2 g in two doses) in children who had (i) unavailable or unknown antibiotic susceptibility results, (ii) double antibiotic resistance to clarithromycin and metronidazole, or (iii) a previous failed therapy.^11^ In a sub‐cohort of *H. pylori*‐infected children with an indication for high‐dose amoxicillin according to guidelines 2016,^11^ only approximately half received the recommended high‐dose regimens. Adverse events occurred in 16% of children receiving high (*N* = 74) versus 11% with standard amoxicillin dose regimens (*N* = 79) (*p* = 0.48) (Supporting Information S1: File S5). Low compliance occurred in 8% receiving high amoxicillin dose (*N* = 74) compared to 15% with standard dose (*N* = 79) (*p* = 0.20). High compared to standard amoxicillin dose regimens reached higher eradication rates in children experiencing adverse events (81% vs. 63%, *p* = 0.03) and in those with excellent compliance (84% vs. 66%, *p* = 0.02) (Supporting Information S1: File S5).

## 4 DISCUSSION

Real‐world data from the EuroPedHp registry provide valuable insights into factors associated with increased risk for lack of monitoring post‐therapy and low compliance. Overall, one in three children did not return for monitoring, underscoring ongoing challenges and significant regional differences addressing barriers to healthcare access in these regions. Our findings highlight the importance of compliance with drug intake for therapy success. We identified several factors that increase the risk of low compliance and impact eradication outcomes in clinical practice, such as recurrent vomiting, having peptic ulcers/erosions, and experiencing adverse events during therapy.

Overall, one in three children did not undergo post‐therapy monitoring, underscoring ongoing challenges in ensuring adequate follow‐up and adherence to treatment guidelines. These findings highlight the need to better understand and address system‐level and patient‐related barriers that impact eradication outcomes in clinical practice.

Monitoring visits and assessing *H. pylori‐*clearance after prescribing treatment^8^, ^9^, ^17^ is considered as standard of care.^7^, ^10^, ^11^, ^12^, ^17^, ^24^ Follow‐up visits allow to assess ongoing symptoms, compliance, and drug‐related adverse events, in addition to testing for treatment success. If testing indicates failed clearance, pros and cons of a second treatment with different regimens should be discussed with patients and caregivers,^15^, ^18^, ^24^ regardless whether symptoms had improved.^19^ Eradicating infection reduces risk of later complications for individuals and spread of resistant strains within the population.^8^, ^9^, ^17^

The 31% rate of missing post‐treatment visits in children with prescribed therapy is higher than the 23.8% observed in our previous paediatric registry 2013–2016 ^21^ and the 6% in the European adult registry (Hp‐EuReg registry).^20^ A Spanish sub‐analysis of the Hp‐EuReg registry identified several reasons for lack of follow‐up, including clinical improvement, side effects, and treatment interruption.^25^ Nonetheless, it remained uncertain whether patients voluntarily abandoned follow‐up visits or if physicians failed to recommend confirmatory testing.^25^ Further reasons for low retesting rates include transitions in patient care between multiple providers, for example, from the specialist to primary care physician, settings, and limited access to care in certain regions.^18^ We found that children with dyspepsia and nausea were less likely to be followed. We speculate that feeling nauseated might hinder patients from taking prescribed drugs, potentially leading to treatment interruptions and missed follow‐up visits. Gastrointestinal bleeding is often sudden and alarming, prompting emergency visits and hospital admissions. After acute stabilisation, patients might fail to return to regular care, especially if facilities are distant. Notably, children without antibiotic susceptibility results were twice likely for loss to follow‐up, while recommending probiotics during therapy were associated with higher rates of monitoring visits. We hypothesize that providing precise individualised instructions with rational arguments to caregivers and patients may improve adherence.

Our analyses confirm that non‐compliance with prescribed medication significantly diminishes the probability of successful eradication and potentially contribute to subsequent emergence of antibiotic resistance.^10^, ^11^, ^15^, ^16^, ^20^, ^26^, ^27^, ^28^ The registry data highlight regional differences, for example, children living in Israel and Türkiye faced nearly seven‐fold higher risk of low compliance compared to those living in Northern/Western Europe. Immigrant children were twice likely to show low compliance indicating language barriers. Moreover, low compliance was significantly associated with frequent vomiting, peptic ulcers or erosions, experiencing side effects during treatment like diarrhoea, vomiting, dyspepsia, nausea, or metallic/disturbed taste, common with clarithromycin therapy. Our results align with findings from adult registry data.^29^, ^30^ Huguet et al. demonstrated that low compliance is related to longer therapy duration and higher antibiotic doses of the prescribed regimen increasing the risk for adverse events and possibly therapy discontinuation.^29^

Non‐adherence is a complex multifaceted problem influenced by patient, family, physician, regimen, and systemic factors.^31^, ^32^ Key challenges include the critical family role in medication administration, issues with medication formulations and palatability, the timing of medication doses and its impact on daily routines.^32^

Written instructions should be provided in the patient's native language. In 2018, the ESPGHAN working group developed a flyer on *H. pylori* infection in an easy to understand language with pictograms for patients and caregivers.^33^ The flyer explains diagnosis, treatment, and common but typically mild and non‐harmful therapy‐related adverse events. It emphasizes the importance of completing the full treatment course and follow‐up visit to confirm successful eradication. The flyer includes a diary for parents/patients to document daily medication intake and adverse events during therapy.

Therapy compliance was better in high compared to standard amoxicillin dose regimens. However, high amoxicillin dose was only recommended by the guidelines 2017 for selected children, those with previous failed therapy or infected with double resistant strains or unknown antibiotic susceptibility.^11^ For unknown reasons, physicians prescribed high dose amoxicillin regimens in only half of this subgroup. Despite possible selection bias and limited sample size, the high amoxicillin dose regimen demonstrated significant benefits for patients with excellent compliance. The most recent ESPGHAN/NASPGHAN guidelines from 2024^12^ take this into account and recommend first line to all children high dose amoxicillin TTT for 14 days considering only clarithromycin, but not metronidazole susceptibility results. While our data indicated good medication compliance in the selected subgroup and within the specific setting of the registry, it remains uncertain whether this would be maintained in broader clinical practice. For these reasons, prospective data from a large number of *H. pylori*‐infected paediatric patients from different countries and settings should evaluate the feasibility, acceptability, compliance, and eradication rates applying these recommendations as first‐line therapy in routine care.

Our study is one of the first to systematically analyse factors associated with loss to follow‐up and low compliance in a large multinational paediatric cohort. Our comprehensive data provide valuable insights into follow‐up challenges in routine clinical care, where physician feedback is often limited. Though our findings are robust and account for several confounders, they should be interpreted with caution. Our study's limitations include observational nature of the data and potential selection bias since participating physicians as members of the *H. pylori* Special Interest Group of ESPGHAN have been more motivated to distribute the flyer to their patients and caregivers to improve compliance. Additionally, physician‐reported compliance may introduce reporting bias. The 8% rate of children reporting non‐compliance may be biased by the high number of children without follow‐up visits.

## 5 CONCLUSION

The registry data including 1263 children with biopsy proven *H. pylori* infection showed 31% lost to follow‐up after prescribed therapy and 8% low compliance indicating room for improvement. Feedback loops between prescribing and follow‐up physicians are essential, particularly in children at increased risk for non‐adherence, to maximize treatment outcome. We strongly support the recommendation to monitor patients 6‐8 weeks post‐treatment by C^13^ urea breath test or monoclonal stool antigen test to ensure successful *H. pylori‐*eradication or to take action if therapy failed.^10^, ^11^, ^12^ Identified risk factors for poor compliance, such as recurrent vomiting, peptic ulcers/erosions, experiencing adverse events during therapy, and clarithromycin containing regimens call for supportive care to mitigate these symptoms. Patients and caregivers should be encouraged to adhere to drug intake, unless symptoms are unbearable. We strongly suggest clinicians invest time in explaining the need of follow‐up visits and medication adherence. The flyer developed by the *H. pylori* working group of ESPGHAN ^33^ and freely available for download in multiple languages may further help to improve compliance and eradication success. (https://www.espghan.org/knowledge-center/education/H-Pylori-Patient-Parent-Guide).

## CONFLICT OF INTEREST STATEMENT

Sibylle Koletzko reports personal honoraria for Adboard or from speaker's fee from AstraZeneca, AbbVie, Nestle Nutrition, Danone, Janssen, Pfizer, Proveca, Sanofi, Takeda, Tillotts, outside the submitted work. Zrinjka Misak reports speakers’ honoraria and travel grants from Sandoz, Ewopharma, GM Pharma, Ferring Pharmaceuticals, and Milsing, outside the submitted work. Martha Taveres reports speakers’ honoraria from Biocodex and Sanofi outside of the submitted work. Nicolas Kalach reports speakers’ honoraria from Danone France, Novalac France, Mead Johnson France, Biofortis France, as well as fees for participating in Advisory Board from Dr Falk, and Sanofi, outside of the submitted work. Alexandra Papadopoulou reports research grants from Abbvie, United Pharmaceuticals, Dr. Falk Pharma GmbH, Takeda, AstraZeneca; speaker's honorariums from Cross Pharmaceuticals, Friesl and Campina, Nestle, Petsiavas, Sanofi Regeneron Pharmaceuticals, SYN Innovation Laboratories, Uni‐Pharma Pharmaceuticals Laboratories S.A; and fees for participating in advisory board of specialty therapeutics outside of the submitted work. Erasmo Miele reports honoraria for advisory boards and lectures from Bioprojet, Dicofarm, Pfizer, and Recordati, none of which are related to this submitted work. The remaining authors declare no conflicts.

## Supporting information

26.01.06_R1_JPGN_Supplementary files_clean.
