# Supplementary material for: Factors related to loss to follow‐up and low compliance in Helicobacter pylori‐infected children: The EuroPedHp Registry
Source: J Pediatr Gastroenterol Nutr. 2026 Feb 24;82(5):1178–92. doi: 10.1002/jpn3.70387 (PMC13150790; doi:10.1002/jpn3.70387)
Supplement: Supplementary file 1 — 26.01.06_R1_JPGN_Supplementary files_clean. [file JPN3-82-1178-s001.docx]

# . Factors related to loss to follow-up and low compliance in *Helicobacter pylori*-infected children: the EuroPedHp Registry

Thu Giang **Le Thi^[[1]](#footnote-1),^^[[2]](#footnote-2)^**, Kallirroi **Kotilea^[[3]](#footnote-3)^**, José **Cabral^[[4]](#footnote-4)^**, Michal **Kori^[[5]](#footnote-5)^**^,^**^[[6]](#footnote-6)^**, Maria Luz **Cilleruelo^[[7]](#footnote-7)^**, Marta **Tavares^[[8]](#footnote-8)^**, Josefa **Barrio^[[9]](#footnote-9)^**, Vaidotas **Urbonas^[[10]](#footnote-10)^**, Matjaž **Homan^[[11]](#footnote-11)^**, Zrinjka **Misak^[[12]](#footnote-12)^**, Nicolas **Kalach^[[13]](#footnote-13)^**, Pedro **Urruzuno^[[14]](#footnote-14)^**, Martina **Klemenak^[[15]](#footnote-15)^**, Andreas **Krahl^[[16]](#footnote-16)^**, Andrea **Chiaro^[[17]](#footnote-17)^**, Josef **Sykora^[[18]](#footnote-18)^**, Meltem **Korkut Ugras^[[19]](#footnote-19)^**, Jan de **Laffolie^[[20]](#footnote-20)^**, Erasmo **Miele^[[21]](#footnote-21)^**, Alexandra **Papadopoulou^[[22]](#footnote-22)^**, Sibylle **Koletzko^1,^^[[23]](#footnote-23)^** for the *Helicobacter pylori* & other Gastritis Special Interest Group of ESPGHAN

**Supplementary files:**

[Supplementary file 1: Countries assigned to four geographical regions (1) 3](#_Toc208504236)

[Supplementary file 2: Flow-chart of the study population 4](#_Toc208504237)

[Supplementary file 3: Factors associated with loss to follow-up among treatment naïve pediatric patients with prescribed therapy for *H. pylori* infection, N=1052 5](#_Toc208504238)

[Supplementary file 4: Factors associated with low therapy compliance among treatment naïve pediatric patients treated for *H. pylori* infection, N=708 6](#_Toc208504239)

[Supplementary file 5: Eradication success in *H. pylori*-infected children of 14-day tailored triple therapy (TTT) using high versus standard dose amoxicillin regimens* modified by adverse events during therapy and therapy compliance. 7](#_Toc208504240)

# Supplementary file 1: Countries assigned to four geographical regions (1)

| **Country regions** | **Countries** |
| --- | --- |
| **Northern/Western Europe** |  |
| Northern Europe | Sweden, Norway, Finland |
| Western Europe | United Kingdom (UK), Ireland, France, Netherlands, Belgium, Germany, Austria, Switzerland, Luxembourg |
| **Southern Europe** | Portugal, Spain, Italy, Greece |
| **Eastern Europe** | Albania, Bosnia, Kosovo, Serbia, Macedonia, Romania, Slovenia, Ukraine, Russia, Lithuania, Poland, Hungary, Czech Republic, Slovakia, Croatia, Moldavia |
| **Asia, Africa, America, and the Middle East** | Angola, Guinea, Somalia, Senegal, Ghana, Cabe Verde, Guinea to Bissau, Madagascar, South Africa, Libya, Morocco, Tunisia, Algeria, Ethiopia, Eritrea, Republic Dominican, China, Mongolia, Thailand, Vietnam, New Guinea, Iran, Iraq, Syria, Egypt, Turkey, Israel, Armenia, Bangladesh, Afghanistan, Kazakhstan, Azerbaijan, India, Nepal, Canada, USA, Colombia, Chile, Peru, Ecuador, Bolivia, Argentina, Brazil, Uruguay, Paraguay, Australia. |

*The countries listed here were compiled by participating hospitals across the demographic survey fields: (1) country of participating centres (country of living), (2) country of birth of the child, (3) country of birth of the mother, and (4) country of birth of the father. This list reflects all entries for the purposes of regional categorization.*

# Supplementary file 2: Flow-chart of the study population


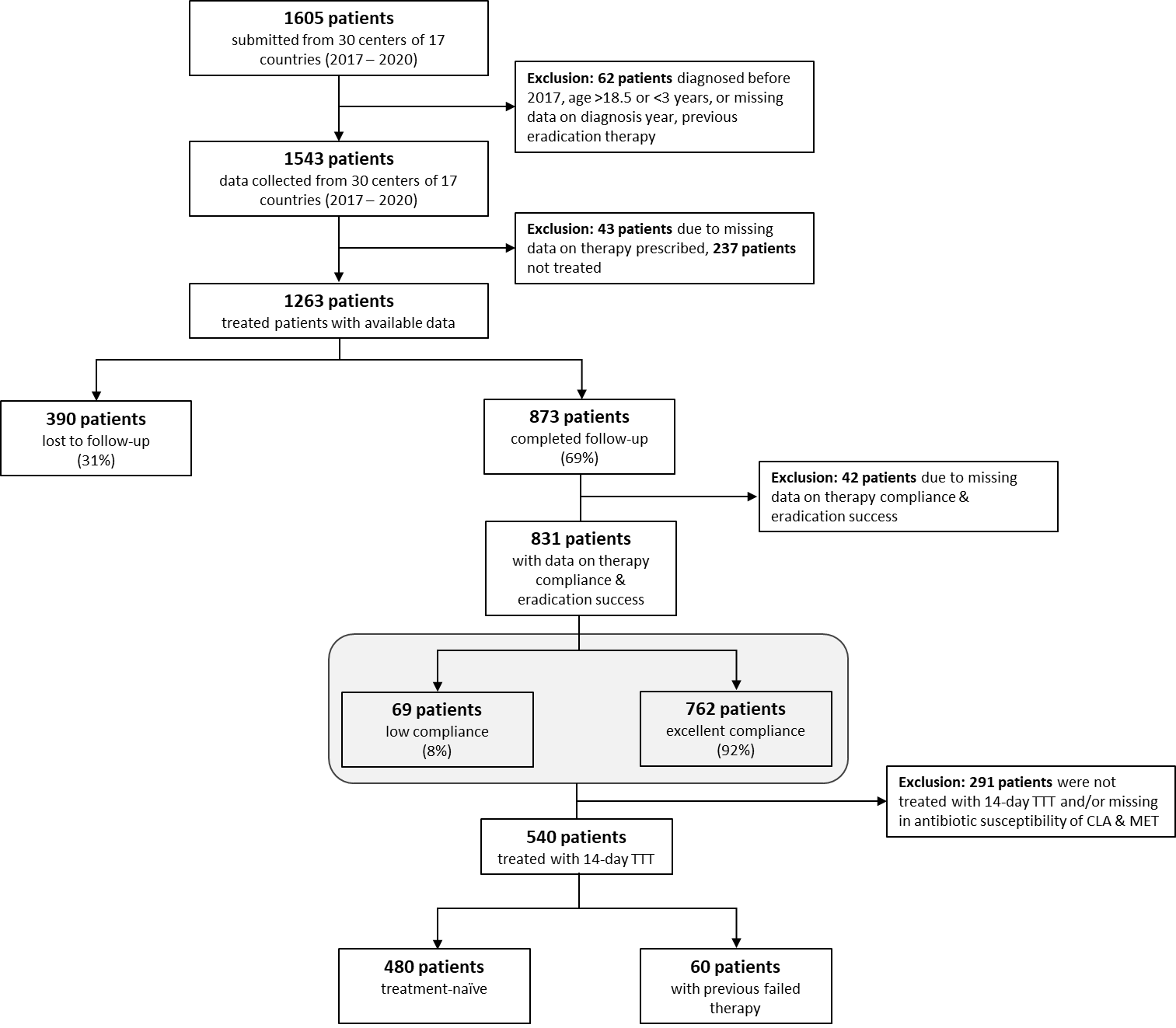


Abbreviations: TTT, tailored triple therapy; CLA, clarithromycin; MET, metronidazole.

# Supplementary file 3: Factors associated with loss to follow-up among treatment naïve paediatric patients with prescribed therapy for *H. pylori* infection, N=1052

Odds ratios (OR) with 95% confidence intervals (95% CI) were obtained from the final multivariable logistic regression adjusted for gender, age, and previous diagnosis of *H. pylori* infection. P-values were determined using the Wald Chi-Square test to assess the significance of the odds ratio (OR).

Abbreviations: GI, gastrointestinal; OR, odd ratio; MET-S/CLA-S: Strains susceptible to both metronidazole and clarithromycin. MET-S/CLA-R: Strains susceptible to metronidazole but resistant to clarithromycin. MET-R/CLA-S: Strains resistant to metronidazole but susceptible to clarithromycin. MET-R/CLA-R: Strains resistant to both metronidazole and clarithromycin.

# Supplementary file 4: Factors associated with low therapy compliance among treatment naïve paediatric patients treated for *H. pylori* infection, N=708

Odds ratios (OR) with 95% confidence intervals (95% CI) were obtained from the final multivariable logistic regression adjusted for gender, age, previous diagnosis of *H. pylori* infection, and previous *H. pylori* eradication. P-values were determined using the Wald Chi-Square test to assess the significance of the odds ratio (OR).

Abbreviations: GI, gastrointestinal; OR, odd ratio; MET-S/CLA-S: Strains susceptible to both metronidazole and clarithromycin. MET-S/CLA-R: Strains susceptible to metronidazole but resistant to clarithromycin. MET-R/CLA-S: Strains resistant to metronidazole but susceptible to clarithromycin. MET-R/CLA-R: Strains resistant to both metronidazole and clarithromycin.

# Supplementary file 5: Eradication success in *H. pylori*-infected children of 14-day tailored triple therapy (TTT) using high versus standard dose amoxicillin regimens* modified by adverse events during therapy and therapy compliance.

**A) Proportion of children experiencing adverse events
B) Eradication rates in children with and without adverse events
C) Proportion of children with excellent and low therapy compliance
D) Eradication rate concerning therapy compliance**

*) The patient cohort comprises children who had (i) negative bacterial culture, unavailable or unknown antibiotic susceptibility results, (ii) double antibiotic resistance to clarithromycin and metronidazole, or (iii) a previously failed therapy and were recommended for a 14-day triple therapy (TTT) regimen using a high-dose amoxicillin according to the updated 2017 guidelines (2).

ER% represents eradication rate (ER) in per cent (%) as the proportion of all patients treated successfully with a confirmed negative test after completed treatment relative to all patients treated in a specific sub-group. P-values were obtained from Fisher-exact-test to determine the significant difference in eradication rate (ER) between groups.

Abbreviations: ns, not significant; *H. pylori*, Helicobacter pylori; AMO amoxicillin; PAC for treatment regimen with proton pump inhibitor, amoxicillin, clarithromycin; PAM for treatment regimen with proton pump inhibitor, amoxicillin, metronidazole.

**References**

1. Le Thi TG, Werkstetter K, Kotilea K, Bontems P, Cabral J, Cilleruelo Pascual ML, et al. Management of Helicobacter pylori infection in paediatric patients in Europe: results from the EuroPedHp Registry. Infection. 2023;51(4):921-34.

2. Jones NL, Koletzko S, Goodman K, Bontems P, Cadranel S, Casswall T, et al. Joint ESPGHAN/NASPGHAN Guidelines for the Management of Helicobacter pylori in Children and Adolescents (Update 2016). J Pediatr Gastroenterol Nutr. 2017;64(6):991-1003.

1. Department of Pediatrics, Dr. von Hauner Children’s Hospital, LMU University Hospital Munich, Germany [↑](#footnote-ref-1)
2. Stiftung Kindergesundheit, c/o Dr. von Hauner Children’s Hospital, LMU University Hospital Munich, Germany [↑](#footnote-ref-2)
3. Université Libre de Bruxelles, Hôpital Universitaire des Enfants Reine Fabiola, Brussels, Belgium [↑](#footnote-ref-3)
4. Child and Adolescent Centre, CUF Tejo Hospital, Lisbon, Portugal [↑](#footnote-ref-4)
5. Pediatric Gastroenterology, Kaplan Medical Centre, Rehovot, Israel [↑](#footnote-ref-5)
6. Faculty of Medicine, Hebrew University of Jerusalem, Jerusalem, Israel [↑](#footnote-ref-6)
7. Pediatrics Department. Gastroenterology Unit. University Hospital Puerta de Hierro Majadahonda, Madrid, Spain [↑](#footnote-ref-7)
8. Division of Pediatrics, Pediatric Gastroenterology Department, Centro Materno Infantil do Norte, ICBAS - Instituto de Ciências Biomédicas Abel Salazar, Porto, Portugal [↑](#footnote-ref-8)
9. Pediatrics Department, Gastroenterology Unit, University Hospital, Fuenlabrada, Madrid, Spain [↑](#footnote-ref-9)
10. Clinic of Children's Diseases of Vilnius University Faculty of Medicine, Vilnius, Lithuania [↑](#footnote-ref-10)
11. Department of Gastroenterology, Hepatology, and Nutrition, University Children’s Hospital, Faculty of Medicine, University of Ljubljana, Ljubljana, Slovenia [↑](#footnote-ref-11)
12. Referral Centre for Pediatric Gastroenterology and Nutrition, Children’s Hospital Zagreb, University of Zagreb School of Medicine, Zagreb, Croatia [↑](#footnote-ref-12)
13. Saint Antoine Pediatric clinic, Saint Vincent de Paul Hospital, Groupement des Hôpitaux de l’Institut Catholique de Lille (GHICL), Catholic University, Lille-France [↑](#footnote-ref-13)
14. Pediatric Gastroenterology Unit. Hospital 12 de Octubre, Madrid, Spain [↑](#footnote-ref-14)
15. Gastroenterology, Hepatology and Nutrition Unit, Department of Paediatrics, University Medical Centre Maribor, Maribor, Slovenia [↑](#footnote-ref-15)
16. Darmstädter Kinderkliniken Prinzessin Margaret, Darmstadt, Germany [↑](#footnote-ref-16)
17. Pediatric Gastroenterology and Endoscopy Unit, Institute Giannina Gaslini, Genoa, Italy [↑](#footnote-ref-17)
18. Department of Paediatrics, Charles University in Prague, Faculty of Medicine in Pilsen, Czech Republic [↑](#footnote-ref-18)
19. Department of Pediatrics, Gastroenterology Hepatology and Nutrition, Yeditepe University Faculty of Medicine, İstanbul, Türkiye [↑](#footnote-ref-19)
20. Department of General Pediatrics and Neonatology, Centre of Child and Adolescent Medicine, Justus-Liebig-University Gießen, Giessen, Germany [↑](#footnote-ref-20)
21. Department of Translational Medical Science, University of Naples Federico II, Naples, Italy [↑](#footnote-ref-21)
22. First Department of Paediatrics, University of Athens, Agia Sophia Children's Hospital, Athens, Greece [↑](#footnote-ref-22)
23. Department of Pediatrics, Gastroenterology and Nutrition, School of Medicine Collegium Medicum University of Warmia and Mazury, Olsztyn, Poland [↑](#footnote-ref-23)
